# Progress in State Medicine

**Published:** 1903-01-03

**Authors:** 


### Progress in State Medicine.

### STATE AND SCHOOLS.

Newsholme [1] remarks that the medical inspection of schools, and particularly of scholars, has taken much deeper root in the United States than in this country. Boston (U.S.A.) inaugurated last December this system. The city is divided into 50 districts, a medical inspector being appointed to each. Each inspector has four or five schools and about 2,000 children under his charge. He is required to visit at their homes, at least twice, all cases of diphtheria and scarlet fever reported in his district. He also visits each school soon after opening of the morning session, the teacher submitting for examination any children who appear to be ill or in any way to need the advice of the inspector. Many cases of sore throat are seen, and in these it is the routine practice to take a culture before sending the child home. Great care is enjoined in refraining from interference with the province of the private physician, the school inspectors not giving professional treatment, but merely pointing out the need for it when it exists. In Boston the inspectors occasionally vaccinate school children. In 1898 the number vaccinated was only 260, but many arms were examined, and 1,142 certificates of vaccination filled up. Children suffering from pediculosis are sent home with a note to the parent stating the nature of the complaint, and requesting that the child be kept out of school for a few days until the disease is cured. Directions are given how to effect the cure, and the child is not readmitted to the school until he brings a certificate to the effect that the treatment has been carried out for three successive days. Recently the School Board for London has been advertising for nurses, one of whose duties is to be the examination of school children's heads for ringworm. To be efficacious this should be succeeded by a further examination by a medical inspector. In Chicago in January 1900, a daily inspection of scholars was begun, there being 50 inspectors who work from 9 a.m. to 12 noon. All children who have been absent for four days or more are required to submit to an examination of their throats before they can return to their class-rooms. In New York in 1898, 139,965 children were examined, and 7,606 excluded. Of these 118 had diphtheria, 328 scarlet fever, 253 measles, 380 chicken-pox, 517 mumps, 1,627 contagious diseases of the eye, 703 contagious diseases of the skin, 3,502 parasites of the head, and 152 parasites of the body. Further medical examination would be most valuable in discovering defective eyesight or hearing, thus enabling the parent to start early treatment. Lea [2] draws attention to the great need there is in Manchester and Salford for more thorough medical inspection of public elementary schools and of scholars. Attendance at school being compulsory it is only just that the school authorities should see that the health of the children does not suffer, and that they should, as far as possible, be protected "from the infectious and loathsome diseases so prevalent in schools." The London School Board hopes that by instructions and pamphlets the teachers themselves will be able to diagnose these complaints, but this is no part of a teacher's work and would be of comparatively little value. He deprecates also the use of trained nurses for this duty as approved by the Board of Education. The expense of the introduction of the American system of daily medical inspection of the scholars would be more than balanced by (1) a diminution in the number of cases of infectious disease; (2) increased school attendance, and (3) improved health of the children. The Manchester School Board estimates that through measles alone the elementary schools of Manchester lose £2,000 per annum. In view of the acknowledged rapid "physical deterioration of the youth of our great cities," it is of high importance to neglect no means of checking what is already a national peril. Collins [3] remarks that it is difficult to say how far the State may safely go in relieving the parents of responsibility, but it is quite certain that a child of the slum, half fed, half blind, half deaf, and with only a half-formed physique, is unable to profit to the full extent by the instruction offered by the State. In aggregating children together in school-rooms it is unavoidable that derangements should occur. Much can be done by building suitable school houses, providing efficient ventilation and warming, and the provision of outdoor covered class-rooms. In Bradford the "State" doctor examined the eyesight of 31,903 children in two years, and found visual defects in 38·3 per cent. of boys and 47·6 per cent. of girls. The greater number of them were hypermetropic. The percentage of defective vision is highest at six years of age—42·3—and gradually decreases up to 14 years, when the percentage is 13·2. In Kingston 37 scholars were absent during the school year, 1900–1901—1,259 days—for various complaints connected with the eyes. The child who does not hear accurately is placed at a disadvantage in the competition with his fellows, and he grows up a dolt, when perhaps timely attention might have made a useful citizen of him. Nineteen cases of defective hearing were recorded in Kingstown, with 122 days' absence. Sixty-two scholars were absent for 539 days for toothache. Those who have had to examine recruits know what a common cause of rejection defective teeth is in persons otherwise suitable for soldiers or stokers. The notifiable diseases are largely spread through schools, more particularly the elementary schools. It is the mild cases that start the epidemics, and it is difficult to see how this is to be avoided except by the slow education of parents. Non-notifiable infectious diseases might with great benefit be made notifiable. With early notification it would be possible to prevent school closure for diseases such as measles. Raising the school age to six or seven would materially reduce the chance of epidemics of these diseases. At Kingstown, ringworm affected 102 scholars, causing 4,358 days' absence. Forty-nine of these scholars were infants, and the total absences were 2,754 days, or 56·2 days per case. Twenty boys were absent 883 days, or 44·1 per case, and 83 girls were absent 721 days, or 21·8 days per case. In nearly every case the children were found to be without medical attendance. The State having made education compulsory is bound to see that the children suffer from no avoidable damage while pursuing their studies. Injury to eyesight or hearing, infectious diseases, etc., are avoidable damage. A little expense will remove the danger, and where that is the case the State is bound to spend the money. Newsholme [4] pleads for the exclusion of children under five years of age from public elementary schools. He refers to the number and percentage of such scholars on the school registers in England and Wales at the end of the school year 1900–1901, and points out that 10·9 per cent. of the total scholars at all ages are under five years of age, 10·2 per cent. are between five and six years of age, while only 4·7 per cent. are over 13 years of age. There are strong hygienic objections to putting children of such tender years to word-building or working patterns with needle and worsted, or, in fact, to any occupation involving trained movements of the muscles of the eye and hand. Near-sightedness much more commonly occurs among such children than among children who do not encounter the eye-strain of school life until they are five or six years old. Any form of school lessons before the age of five years is educationally injurious, and is a waste of public money. For children under five the evil effects of aggregation are more marked than for older children, showing themselves not only in the excess of infectious diseases, but also in excess of diseases of the respiratory organs, and in general ill-health. Among the well-to-do classes, the proportion who go through life without having suffered from any of the infectious diseases of childhood is considerable; among the classes whose children attend public elementary schools, this proportion is very small. Admitting that these schools do not account for the whole of the difference, the proportion escaping in the latter would, undoubtedly, be greatly increased by forbidding school attendance before the sixth year of life, and many thousands of lives, now unnecessarily lost, would be saved. The managers of a school, at Martin's suggestion,[5] had every scholar medically examined on returning from the Christmas holidays. Two children were found to be desquamating after scarlet fever, 10 had impetigo contagiosa, one had scabies, 92 had pediculi in their heads, making 43 per cent. suffering from some contagious condition. Most of the above were excluded: three months later four parents of children in the last group were prosecuted for the non-attendance of their children and fined in spite of the fact that they were excluded because of the parasitic state of their heads. It is monstrous that children who are clean should be compelled to attend school with those who are not. A difficulty arises that if "dirty" heads are to be an excuse for not attending school, parents will avail themselves of such a condition to keep their children at home and so evade the law. The remedy for this may be found in the Prevention of Cruelty to Children Act, 1894, for to allow a child's head to remain "dirty" is likely to cause unnecessary suffering and injury to health. McDougall [6] believes that it would be of immense service to public health if every girl attending elementary schools had as part of her education during the last year of her school life to learn practical lessons in cooking simple foods and the details of cleanly and economical use of the fireplace and household utensils. At the same time the methods of cleansing the house could be easily taught. It would be a profitable expenditure of educational finance in State-aided schools that food materials should be purchased of sufficient quantity to give one good meal in the day to the most needy of the scholars, to be cooked by the elder girls under efficient supervision. Such training of girls before leaving school would also have a good effect in their homes whilst still scholars. Early this year [7] the School Board for London appointed eight oculists to pay 200 visits to schools under the control of the Board with the object of testing the vision of the children. A report has been presented of the work done up to the beginning of the summer vacation beginning July 23rd. Each child, after examination, is presented with a card to be taken home to the parents, on which advice as to treatment is written when necessary. Serious visual defects were found in 8 per cent. of the boys and 11 per cent. of the girls. These proportions seemed to be maintained throughout school life. Taking into consideration the various groups of schools, it may be said that the percentage of visual defects varies enormously, being small in the better-class districts and greater among the poor. Certain points are suggested as to the effect of eye work—for example, sewing among girls, and habits of near working induced by the academic methods still prevalent in many infant schools. So far the results in 17,245 children have been recorded.

[1] Practitioner, March 1902. [2] Public Health, July 1902. [3] Ibid. [4] Ibid. [5] Ibid. [6] Brit. Med. Jour., Aug. 16, 1902. [7] Brit. Med. Jour., Oct. 25, 1902.

